# Efficacy of lumbar decompression under large-channel spinal endoscope in elderly patients with segmental lumbar spinal stenosis

**DOI:** 10.1186/s13018-023-04389-x

**Published:** 2024-01-03

**Authors:** Fei Zhang, Dandan Ye, Wei Zhang, Yapeng Sun, Lei Guo, Jiaqi Li

**Affiliations:** 1 Department of Spine Surgery, The Third Hospital of Hebei Medical University, No.139 Ziqiang Road, Qiaoxi District, Shijiazhuang, 050000 Hebei China; 2 Orthopaedic Institute, The Third Hospital of Hebei Medical University, Shijiazhuang, Hebei China

**Keywords:** Large-channel spinal endoscopic technology, Segmental lumbar spinal stenosis, Elderly

## Abstract

**Objective:**

The present study was conducted with an attempt to explore the overall efficacy of large-channel spinal endoscopy technology in elderly patients with segmental lumbar spinal stenosis.

**Methods:**

We included a total of 68 elderly patients with segmental lumbar spinal stenosis in our hospital from February 2021 to March 2023. The participants were randomly and equally distributed into the study group and the control group using a random number table method. The control group received the open lumbar decompression surgery, and the study group received the lumbar decompression under large-channel spinal endoscopy technology. We compared the surgical conditions of the two groups, including pain level, Oswestry Disability Index (ODI) score, and Japanese Orthopedic Association (JOA) score before surgery, 1 week after surgery, 3 months after surgery, and 1 year after surgery. In addition, we compared the efficacy and adverse reactions 1 year after surgery between the two groups.

**Results:**

Our findings revealed that the operation time, intraoperative blood loss, postoperative drainage volume, and hospital stay in the study group were significantly lower than those in the control group (*p* < 0.05). There was no statistically significant difference in the degree of pain between the two groups before surgery (*p* > 0.05), and the pain intensity of the study group was significantly lower than that of the control group at 1 week, 3 months, and 1 year after surgery (*p* < 0.05). Similarly, preoperative ODI and JOA scores were not significantly different between the two groups (*p* > 0.05), while they were significantly lower in the study group than those in the control group at 1 week, 3 months, and 1 year after surgery (*p* < 0.05). Before surgery, no significant difference was seen in therapeutic efficacy between the two groups (*p* > 0.05), whereas the efficacy was remarkably improved in the study group comparing to the control group at 1 week, 3 months, and 1 year after surgery (*p* < 0.05). All patients in this study were followed up for 10 to 16 months, with an average of 13.29 ± 1.28 months. The incidence of adverse reactions in the study group was significantly lower than that in the control group (*p* < 0.05).

**Conclusions:**

Large-channel spinal endoscopy technology exerted promising results in elderly patients with segmental lumbar spinal stenosis, in terms of reducing the surgical time, intraoperative bleeding, postoperative drainage volume, and hospital stay. The approach also alleviated pain, reduced ODI and JOA scores, and restored lumbar function, with decreased incidence of adverse reactions, thereby promoting patient recovery. It is considered valid for wide clinical application.

## Introduction

With society aging, the incidence of lumbar spinal stenosis has been increasing with the passage of time [1]. According to statistics, the prevalence of chronic pain among the elderly in China is 60.2%, with the lower limbs being the most commonly affected part, followed by the lumbosacral and neck areas (29.7%). The disease is highly associated with the repeated lower limb and lumbosacral pain in elderly patients, with radiographic manifestations of widespread degeneration of multiple lumbar intervertebral discs and secondary spinal stenosis [2]. Unlike ordinary disc herniation, the responsible segment is often unclear and the symptoms and signs are atypical. Surgical treatment can effectively reduce the symptoms of nervous system compression, among which the common treatment methods include laminectomy under general anesthesia or bone graft fusion and internal fixation after decompression [3, 4]. Traditional open surgery, which requires interbody fusion and internal fixation, causes extensive damage to the posterior column structure of the spine. In addition, elderly patients often have underlying diseases and poor body organ function, so open surgery makes them likely to have a variety of postoperative complications, such as large surgical trauma, long postoperative bed rest, and slow recovery [5]. In recent years, limited decompression, a concept recognized by many spinal surgeons, has gained popularity, and the application of percutaneous spinal endoscopy, a mature and precise minimally invasive spinal surgery technology, has gradually expanded in the treatment of lumbar spinal stenosis [6, 7]. However, traditional endoscopic systems have low efficiency in handling bony stenosis structures, resulting in prolonged surgical time and increased surgical risk. Based on our previous research, large-channel spinal endoscopy technology, which conforms to the trend of modern minimally invasive, precise operations with high efficiency, is suitable for elderly patients who cannot tolerate major surgery and require rapid postoperative recovery. This technique has a satisfactory therapeutic effect with fewer complications. Due to the limited number of previous studies, this study aims to conduct a randomized controlled study with an expanded sample size to further clarify the application value of large-channel spinal endoscopy in elderly patients with segmenting lumbar spinal stenosis.

## Materials and methods

### General information

We included a total of 68 elderly patients with segmental lumbar spinal stenosis in our hospital from February 2021 to March 2023. The participants were randomly and equally distributed into the study group and the control group using a random number table method. The control group received the open lumbar decompression surgery, and the study group received the lumbar decompression under large-channel spinal endoscopy technology. There was no drop-out during the follow-up after the patients were enrolled. The study group consisted of 14 males and 20 females, aged ranging from 75 to 89 years, with an average of 81.93 ± 11.98 years. The control group consisted of 12 males and 22 females, aged ranging from 76 to 88 years, with an average of 80.98 ± 11.73 years. The general information between two groups of patients was comparable (*p* > 0.05). The experimental protocol was developed according to the Declaration of Helsinki ethical guidelines and approved by the Ethics Committee of the Third Hospital of Hebei Medical University. Written informed consent was obtained from the participants.

### Selection criteria

#### Inclusion criteria

1. Age ≥ 75 years old;
2. Main symptom of unilateral lower limb radiation, alongside persistent pain or intermittent claudication, with or without low back pain (pain severity of low back was less than leg pain);
3. Imaging showing at least two segments of lumbar spinal stenosis, without significant lumbar instability or spondylolisthesis;
4. The symptoms, signs, and imaging showed multiple lumbar segmental damage;
5. Ineffective or recurrent episodes after at least 6 months of conservative treatment;
6. Both parties signed informed consent forms.

#### Exclusion criteria

1. Mental disorders or lack of cooperation;
2. Multisegmental lumbar spinal stenosis with severe lumbar degenerative scoliosis or developmental deformities;
3. Clear presence of lumbar spondylolisthesis or instability;
4. Elderly patients with lower back and leg pain caused by trauma, tumors, tuberculosis, severe osteoporosis, etc.;
5. Non lumbar-associated diseases, such as pelvic and lower limb joint diseases;
6. Patients accompanying spinal tumors or infections;
7. Previous history of spinal surgery;
8. Patients who withdrew midway.

## Methods

The control group received open lumbar decompression. The patient was placed in prone position and underwent general anesthesia. The choice of a unilateral or bilateral approach depends on the patient's symptoms and whether the imaging findings are unilateral or bilateral. Fluoroscopic positioning by G-arm machine. Decompression was performed by incision of the affected laminae, medial to the root of the spinous process and lateral to the lateral facet of the articular process. The hyperplasia of the ligamentum flavum was removed. Expose the dural sac and nerve roots. Protect the nerve vessels. The thickened cortical bone inside the facet joint was removed, and the nerve root canal and recess of the affected side were explored to decompress the nerve root of the affected side. After complete decompression and no bleeding, indwelling negative pressure drainage tube was placed and layer-by-layer suture was completed.

**Fig. 1:**
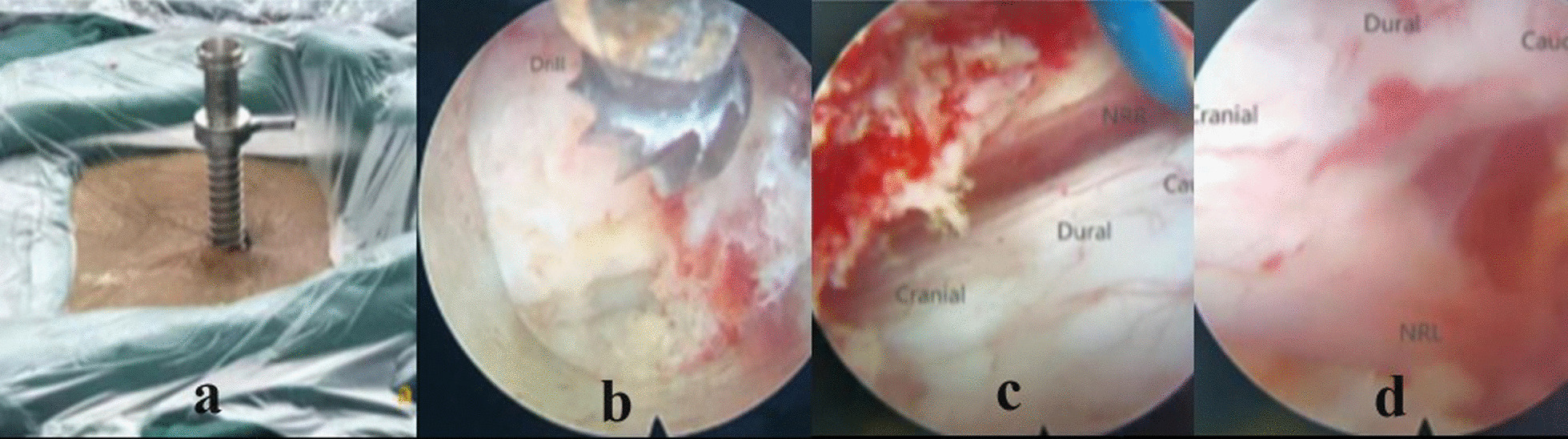
Intraoperative image of a typical lumbar spinal stenosis patient. **a** for the establishment of large channels; **b** for bone decompression under microscope grinding and drilling; **c** indicates adequate contralateral decompression at the L4/5 segment; **d** indicates adequate ipsilateral decompression at the L4/5 segment

The study group underwent large-channel spinal endoscopy technology with the same anesthesia and posture as the control group. The operating table was adjusted to allow the patient to flex their hips, knees, and waist as much as possible to widen the intervertebral space. The target intervertebral space was determined under C-arm fluoroscopy, and routine disinfection and draping were performed. A long incision of approximately 1.2 cm was made at a location around 0.5 cm adjacent to the spinous process on the side with severe symptoms or a high degree of stenosis in the target intervertebral space. The skin, subcutaneous tissue and fascia were cut, and a pen-shaped guide rod and tongue-shaped sleeve were inserted to probe the lower edge of the upper vertebral plate of the target space. After confirming the proper anchoring position through C-arm fluoroscopy (Fig. 1a), the lower margin of the upper and upper laminae of the lower vertebral bodies was identified under endoscopy, and the bony decompression of the ipilateral laminae was performed with endoscopic dynamic drilling (Fig. 1b). The decompression sequence was the lower laminae margin of the upper vertebral body and the upper laminae margin of the lower vertebral body. The medial lamina was treated first and the lateral lamina was treated later. After the ipsilateral bone decompression was completed, nucleus pulposus forceps and radiofrequency were used to remove soft tissue and stop bleeding. Under the direct vision of the endoscope, the bone at the lower margin of the upper laminae exposed the upper stop of the ligamentum flavum, the bone at the upper margin of the lower laminae on the same side exposed the lower stop of the ligamentum flavum, and the bone at the inner margin of the lower articular process on the same side opened the lateral recess. Adjust the position and angle of the sleeve, and remove the base of the lower third of the spinous process and the middle of the spinous process using a half-tooth visible ring saw and a spear pliers. The hyperplasia and cohesive bone in the ventral and upper and lower articular processes of the contralateral upper and lower laminae were removed by the "over the top" of the dynamic system under the gun forceps or microscope. Open the upper and lower ligamentum flavum and expose the lateral recess, remove the ligamentum flavum with nucleus pulposus forceps and blue scissors, and check whether the dural sac and bilateral nerve roots are loosened. When necessary, the "intrathecal sheath" was inserted to perform dural and bilateral nerve root ventral decompression (Fig. 1c). Radiofrequency hemostasis was sufficient. When no active bleeding was detected and the nerve dural sac was loosened, the endoscope was withdrawn, negative pressure drainage balls were placed, and the incision was closed.

Postoperative treatment and rehabilitation procedures: Antibiotics were used to prevent infection before induction of anesthesia, and low molecular weight heparin was used for postoperative anticoagulation in patients at high risk of lower extremity deep vein thrombosis. On the first day post-surgery, patients should undergo straight leg lifting and ankle pump exercises to enhance active flexion and extension of the toe joint. The catheter can be removed on the second day post-surgery to assist with getting out of bed and movement. From 3–5 days after surgery, lumbar and dorsal muscle function exercises with slow movements should be performed. One week after surgery, waist and back muscle function exercise should be performed. Patients should supplement their diet with water and electrolytes daily and consume a digestible protein-rich diet to replenish the protein lost during and after surgery drainage.

### Observation indicators

#### Pain intensity

The Visual Analog Scale (VAS) [8] is a tool to subjectively determined the pain intensity experience by individuals. In this study, we applied this scale to evaluate the intensity of low back pain. The score of no pain is 0, increasing in order, with the extreme pain being 10. The corresponding score was selected based on the patient’s intensity of back pain.

#### ODI score

The Oswestry Disability Index (ODI) [9] is composed of 10 questions about the impact of low back pain on daily life, including pain intensity, personal care, lifting, walking, sitting, standing, sleeping, sexual activity, social life, and traveling, with a score of 0–5 points for each question. The high or low score represents the high or low severity of the dysfunction.

#### JOA score

The Japanese Orthopedic Association (JOA) [10] scoring systems were utilized in this study to evaluate patients’ clinical symptoms (motor disorders, sensory disorders, straight leg elevation disorders, with 0–2 points for each item), with a highest possible total score of 29 points. The degree of good lumbar function of the patient is directly proportional to the score.

#### Sagittal balance of the lumbar spine and lumbar—pelvic parameters

**Fig. 2:**
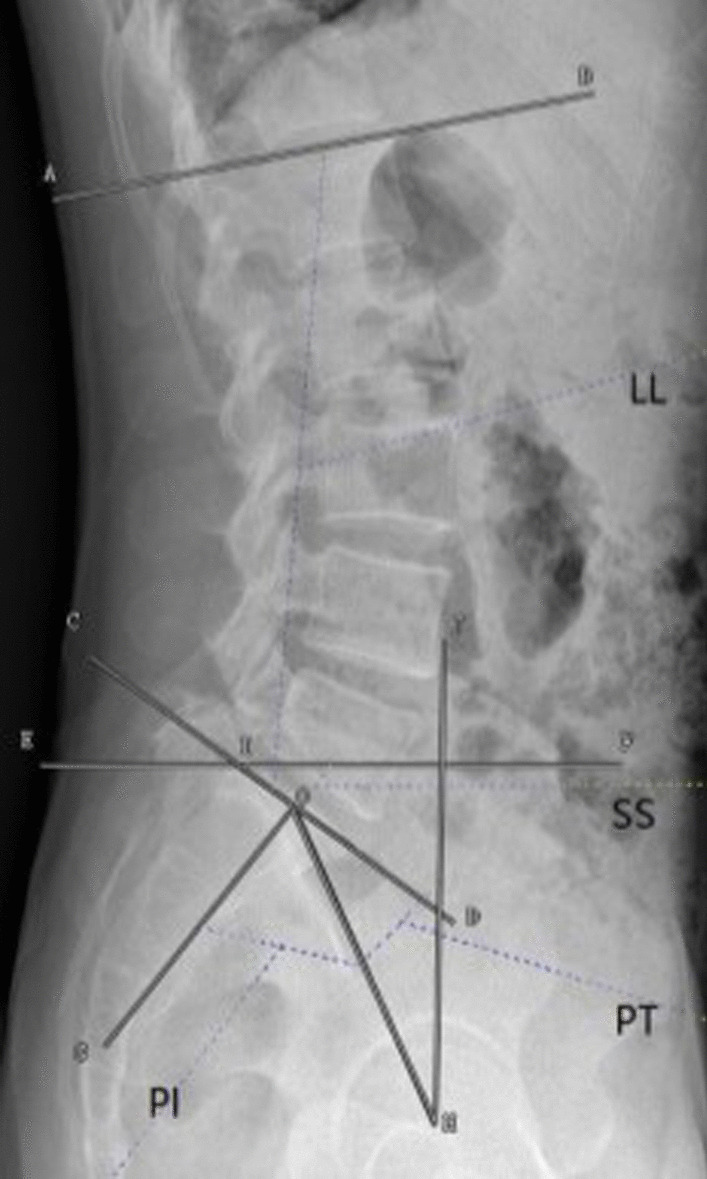
Sagittal balance of the lumbar spine and lumbar—pelvic parameters. Indicators include pelvic incidence angle (PI), lumbar lordosis angle (LL), intervertebral height (DH), pelvic inclination angle (PT), oblique angle (SS), L1 plumb line distance from S1 (LASD)

Indicators include pelvic incidence angle (PI), lumbar lordosis angle (LL), intervertebral height (DH), pelvic inclination angle (PT), oblique angle (SS), L1 plumb line distance from S1 (LASD) (Fig. 2).

#### Therapeutic efficacy

Clinical efficacy was evaluated using the improved Macnab standard [11] one year after surgery. Excellent: Symptoms completely disappeared, and the original work and life are restored; Good: Mild symptoms, mild activity restriction, no impact on work and life; Medium: Symptoms reduced, limited activity, affecting normal work and life; Poor: There is no difference or even worsening before and after treatment.

#### Adverse reactions

The adverse reactions that occurred during the treatment process were monitored and recorded.

### Statistical analysis

SPSS 21.0 statistical software was applied for data processing. Measurement data conforming to normal distribution were expressed as mean ± standard deviation ($\overline{x}$ ± *s*). T-test or one-way ANOVA of two independent samples was used for inter-group comparison. On the other hand, data failing to conform to the normal distribution were represented by the median (quartile) [M (P25, P75)], and Mann–Whitney analysis or Kruskal–Wallis test was used for inter-group comparison. The counting data was represented by the number of cases (percentage) (n, %), and the comparison between groups was performed using chi-square test or Fisher’s exact probability method. The difference was considered statistically significant when *p* < 0.05.

## Results

### Comparison of general information

There was no significant difference in gender, age, smoking, body mass index, and comorbidities between the two groups (*p* > 0.05), as seen in Table 1.

**Table 1:** Comparison of general information

Parameter	Study group (*n* = 34)	Control group (*n* = 34)	*t*/*χ*^2^	*P*
Gender (*n*, male)	14	12	0.249	0.618
Age (year)	81.93 ± 11.98	80.98 ± 11.73	0.329	0.742
Smoking	8	6	0.249	0.618
BMI (kg/m^2^)	23.76 ± 2.31	23.69 ± 2.17	0.129	0.898
Comorbidity				
Diabetes	9	12	0.621	0.431
Coronary heart disease	6	8	0.361	0.549
Hypertension	2	3	0.216	0.642

BMI, Body Mass Index

### Comparison of surgical conditions

The surgical time, intraoperative bleeding volume, postoperative drainage volume, and hospital stay in the study group were significantly lower than that in the control group (*p* < 0.05, Table 2).

**Table 2:** Comparison of surgical conditions ($\overline{x}$ ± *s*)

Parameter	Study group (*n* = 34)	Control group (*n* = 34)	*t*/*χ*^2^	*p*
Surgical time (min)	112.81 ± 23.28	132.39 ± 22.18	3.551	< 0.001
Intraoperative bleeding volume (mL)	20.17 ± 3.91	100.18 ± 18.93	24.136	< 0.001
Postoperative drainage volume (mL)	103.28 ± 19.76	139.91 ± 19.87	7.622	< 0.001
Hospital stay (d)	6.18 ± 1.01	11.93 ± 1.31	20.269	< 0.001

### Comparison of pain intensity

Before surgery, there was no significant difference in the pain intensity between the two groups (*p* > 0.05); at 1 week, 3 months, and 1 year after surgery; however, evidently lower pain intensity was found in the study group compared to the control group (*p* < 0.05) (Table 3).

**Table 3:** Comparison of pain levels ($\overline{x}$ ± *s*, points)

Time	Study group (*n* = 34)	Control group (*n* = 34)	*t*/*χ*^2^	*p*
Before surgery	6.91 ± 1.98	6.87 ± 1.87	0.086	0.932
1 week after surgery	2.01 ± 0.32	3.08 ± 0.19	− 16.765	< 0.001
3 months after surgery	1.18 ± 0.21	2.17 ± 0.24	− 18.101	< 0.001
1 year after surgery	0.87 ± 0.19	1.76 ± 0.22	− 17.853	< 0.001

### Comparison of ODI and JOA scores

Before surgery, the ODI and JOA scores did not differ between the two groups (*p* > 0.05), while they were significantly lower in the study group than those in the control group at 1 week, 3 months, and 1 year after surgery (*p* < 0.05), as laid out in Table 4.

**Table 4:** Comparison of ODI and JOA scores ($\overline{x}$ ± *s*, points)

Indicator	Time	Study group (*n* = 34)	Control group (*n* = 34)	t	P
ODI	Before surgery	39.71 ± 4.39	39.37 ± 4.76	0.306	0.761
	6 months after surgery	17.38 ± 2.18	23.91 ± 2.09	− 12.608	< 0.001
	1 year after surgery	9.71 ± 1.82	14.29 ± 1.92	− 10.095	< 0.001
JOA	Before surgery	11.98 ± 2.19	12.32 ± 2.07	− 0.658	0.513
	6 months after surgery	25.19 ± 2.76	20.12 ± 2.34	8.171	< 0.001
	1 year after surgery	27.91 ± 2.37	23.28 ± 2.09	8.544	< 0.001

ODI, Oswestry Disability Index; JOA, Japanese Orthopedic Association

### Sagittal balance of the lumbar spine and lumbar—pelvic parameters

There were no significant differences in DH, LASD, SS, PT and LL between the two groups before and after surgery (*p* > 0.05), as seen in Table 5.

**Table 5:** Sagittal balance of the lumbar spine and lumbar—pelvic parameters

Indicator	Time	Study group (*n* = 34)	Control group (*n* = 34)	t	P
DH	Before surgery	6.53 ± 0.37	6.49 ± 0.41	0.422	0.674
	3 months after surgery	7.76 ± 0.43	7.63 ± 0.39	1.306	0.196
LASD	Before surgery	20.37 ± 2.42	20.41 ± 0.45	0.379	0.706
	3 months after surgery	39.48 ± 3.48	38.99 ± 3.43	0.585	0.561
SS	Before surgery	32.37 ± 2.28	32.29 ± 2.32	0.143	0.886
	3 months after surgery	30.23 ± 2.22	30.68 ± 2.23	0.834	0.407
PT	Before surgery	17.38 ± 2.19	17.33 ± 2.25	0.093	0.926
	3 months after surgery	18.73 ± 2.23	18.21 ± 2.21	0.966	0.338
LL	Before surgery	42.87 ± 2.76	42.92 ± 2.73	0.075	0.940
	3 months after surgery	36.65 ± 2.71	36.86 ± 2.69	0.321	0.750

PI, pelvic incidence angle; LL, lumbar lordosis angle; DH, intervertebral height; PT, pelvic inclination angle; SS, oblique angle; LASD, L1 plumb line distance from S1

### Comparison of therapeutic efficacy

**Fig. 3:**
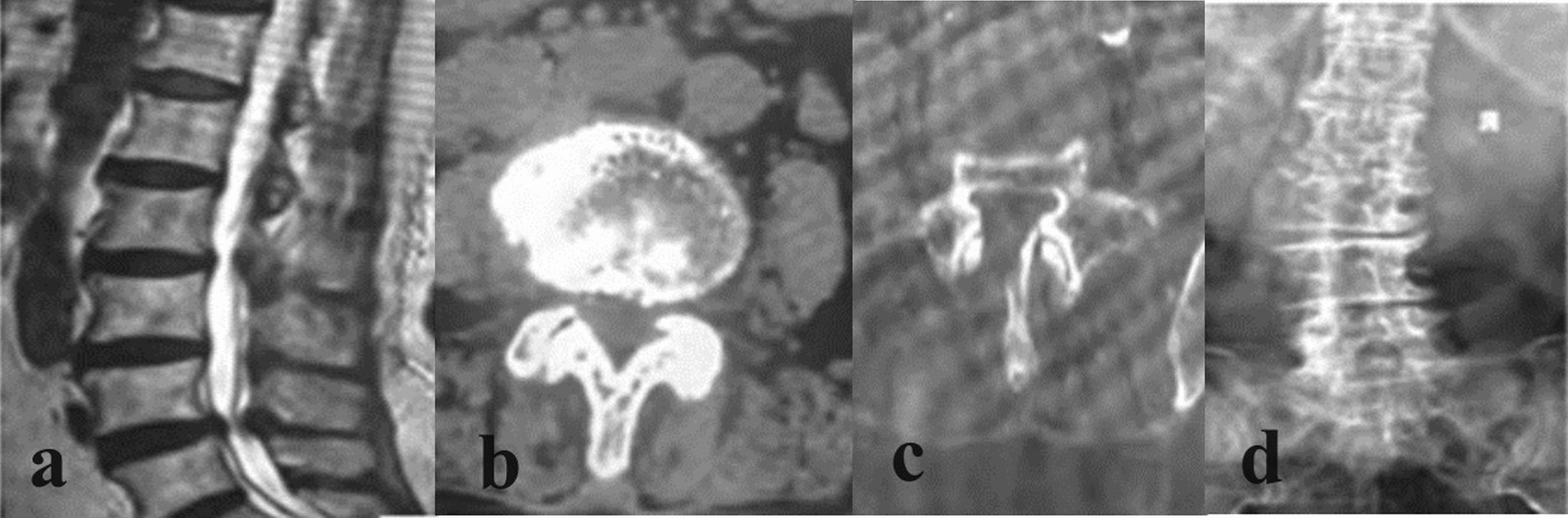
CT and MRI images of typical lumbar spinal stenosis patients before and after operation in study group. An 80-year-old female patient with L4-5 lumbar spinal stenosis was treated by endoscopic technique of large spinal channel.** a** and** b** were preoperative MRI, showing L4-5 lumbar spinal stenosis.** c** indicates adequate decompression of the spinal canal on MRI.** d** showed no lumbar instability on CT 1 month after surgery

**Fig. 4:**
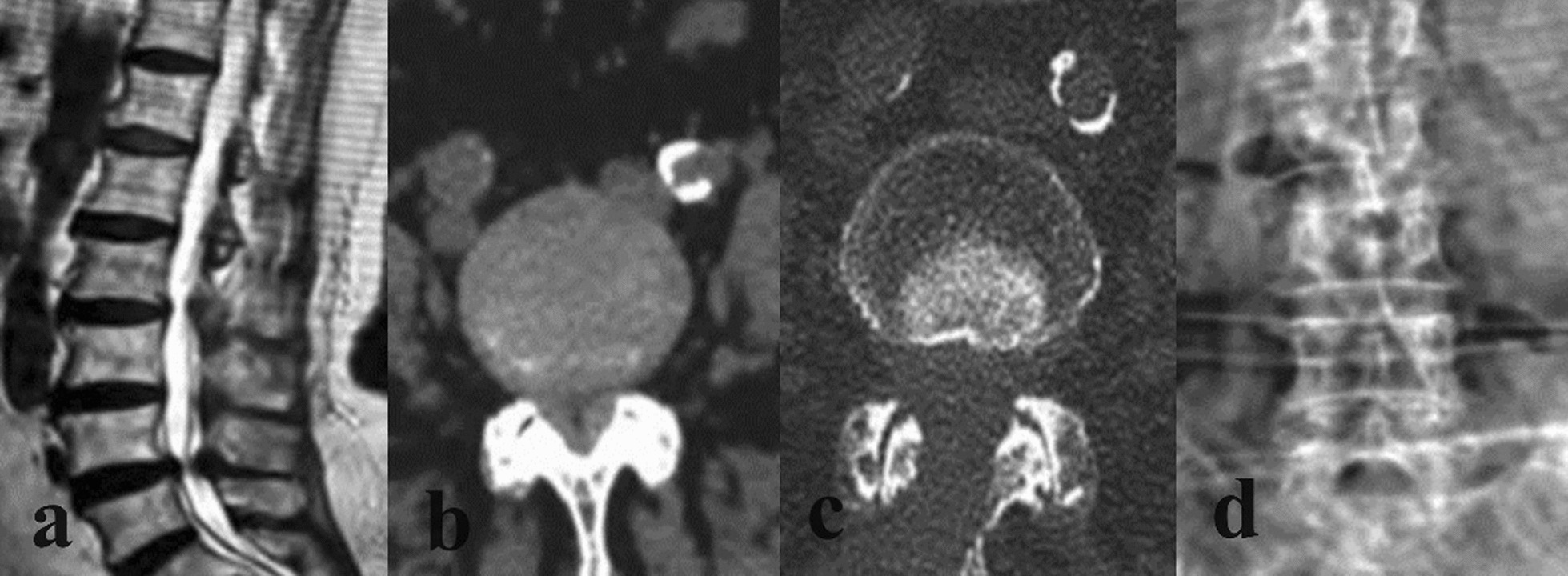
CT and X-ray images of typical lumbar spinal stenosis patients before and after operation in control group. An 84-year-old female patient with L4-5 lumbar spinal stenosis was treated with open decompression.** a** and** b** showed preoperative CT, showing L4-5 lumbar spinal stenosis.** c** shows adequate decompression of the spinal canal on CT.** d** shows the X-radiographs 1 month after surgery, with no lumbar instability

Before surgery, no significant difference was seen in therapeutic efficacy between the two groups (*p* > 0.05), whereas the efficacy was remarkably improved in the study group when comparing to the control group at 1 week, 3 months, and 1 year after surgery (*p* < 0.05), as seen in Table 6 (Figs. 3, 4).

**Table 6:** Comparison of therapeutic efficacy ($\overline{x}$ ± *s*, points)

Indicator	Study group (*n* = 34)	Control group (*n* = 34)	*t*/*χ*^2^	*p*
Excellent	15	9		
Good	16	15		
Medium	2	3		
Poor	1	7		
Total efficacy	33()	27()	5.111	0.024

### Comparison of adverse reaction rates

All patients in this study were followed up for 10 to 16 months, with an average of 13.29 ± 1.28 months. The incidence of adverse reactions in the study group was significantly lower than that in the control group (*p* < 0.05) (Table 7).

**Table 7:** Comparison of adverse reaction incidence ($\overline{x}$ ± *s*, points)

Indicator	Study group (*n* = 34)	Control group (*n* = 34)	*χ*^2^	*p*
Infection	0	2		
Dural laceration	0	2		
Numbness in the lower extremities	1	3		
Total incidence	1	6	2.981	0.046

## Discussion

Lumbar spinal stenosis is a prevalent degenerative disease of the lumbar spine that affects elderly individuals. The pathological changes are mainly due to the narrowing of the “disc yellow space” between the intervertebral disc and the small zygapophysial joint. The small articular process undergoes proliferation and cohesion, and the ligamentum flavum experiences hypertrophy, often accompanied by adhesion of the dural sac or nerve root [12]. Due to the stenosis of the spinal canal or nerve root canal, this compression of nerves and blood vessels within the spinal canal causes neurological dysfunction characterized by intermittent claudication. The pathological basis and clinical characteristics of elderly patients with lumbar spinal stenosis primarily involve degenerative changes such as hyperplasia and cohesion of the small articular process and hypertrophy of the ligamentum flavum. Long-term low back pain leads to poor stress on the lumbar spine and compensatory hyperplasia. The disease onset is often slow, with a prolonged course. Most patients have a longer degenerative segment, while some have accompanying changes such as lumbar spondylolisthesis, scoliosis, and lumbar instability. The symptoms of nerve injury often do not correspond to imaging findings, and the location of the responsible segment may be unclear. Elderly patients with varying degrees of comorbidities and severe underlying medical conditions have limitations in treatment, leading to a significant increase in surgical risk and postoperative complications. Additionally, elderly patients with multiple organ decline and complex perioperative management are prone to adverse reactions such as incision infection or poor healing during open surgery, significantly affecting postoperative efficacy [13]. For surgical treatment of elderly patients with lumbar spinal stenosis, it is necessary to thoroughly decompress and maintain spinal stability while also considering the patient’s overall condition, shortening surgical time and reducing surgical trauma.

Full endoscopy technology has undergone continuous advancement, and it can now achieve targeted and precise decompression, maximize spinal stability, and reduce surgical trauma. There are numerous literature reports both domestically and internationally suggest that full endoscopy, as an emerging minimally invasive technique for the spine that has numerous advantages such as high safety, minimal bleeding, minimal postoperative scars, minimal nerve adhesions, minimal impact on posterior spinal stability, and fast recovery, all of which can be achieved under local anesthesia [7]. Khalifeh et al. [14] revealed that minimally invasive lumbar intervertebral fusion through intervertebral foramen can effectively treat patients with lumbar spinal stenosis. Chen et al.’s study indicated that minimally invasive surgery is a feasible method for treating elderly lumbar spinal stenosis, resulting in shorter surgical time, less bleeding, shorter hospital stay, and fewer complications [15]. The results of this study showed that the surgical time, intraoperative bleeding volume, postoperative drainage volume, and hospitalization time of the study group were lower than those of the control group (*p* < 0.05), indicating that large-channel spinal endoscopy technology was able to shorten the surgical time of elderly patients with segmental lumbar spinal stenosis, reduce intraoperative bleeding volume, postoperative drainage volume, and hospitalization time, with improved recovery. Although pain symptoms are a natural bodily reaction with a certain defensive and protective effect, acute pain following orthopedic surgery is often severe and can lead to various complications. It can also trigger negative emotions in patients, which can affect the recovery process. In addition, pain can prevent patients from getting sufficient sleep and rest, making the choice of surgical method a crucial factor in managing postoperative pain [16]. Klingler et al. [16] have identified minimally invasive surgery as a promising technique for treating spinal stenosis that can effectively alleviate the pain level in patients. The results of this study showed that lower pain intensity was found in the study group than the control group at 1 week, 3 months, and 1 year after surgery (*p* < 0.05), indicating that large-channel spinal endoscopy technology could effectively reduce the pain levels in elderly patients with segmental lumbar spinal stenosis.

The ODI scale has been widely used aboard for over 20 years to evaluate the efficacy of conservative treatment in spinal surgery. It has high validity and reliability and is regarded as the gold standard for assessing treatment effectiveness [17]. Gao et al.’s study reported that minimally invasive surgery can effectively treat lumbar spinal stenosis and improve patients’ ODI scores [17]. Similarly, the study by Awaya et al. demonstrated that minimally invasive micro laminectomy can effectively improve the JOA score in patients with lumbar spinal stenosis [18]. Based on our study, the ODI and JOA scores in the study group were lower than those in the control group at 1 week, 3 months, and 1 year after surgery (*p* < 0.05), suggesting that large-channel spinal endoscopy technology was capable of effectively reducing the ODI score and JOA score in elderly patients with segmental lumbar spinal stenosis, as well as restoring lumbar function. According to Mekhail et al. [12], minimally invasive lumbar spine decompression has been proved to be a safe and effective treatment for patients with lumbar spinal stenosis.

The reduction or disappearance of the normal lumbar lordosis angle can cause compensatory or decompensated balance of the sagittal plane sequence of the lumbar spine, presenting with persistent low back pain and muscle fatigue [19]. Some studies have shown that lumbar lordosis angle is a very important evaluation parameter of spinal sagittal balance and an important reference index in the surgical treatment of correcting lumbar spinal stenosis [19]. According to the studies of Park et al., increasing the height of the focal intervertebral space and improving the lumbar lordosis angle can increase the mechanical gravity of the anterior longitudinal ligament and reduce the strain [20]. It can improve the interbody fusion rate and reduce the degeneration of adjacent vertebral segments. Therefore, it is very necessary to analyze the parameters of the lumbar sagittal plane by X-ray imaging before the lumbar spine, so as to guide the accurate reconstruction of the lumbar lordotic curve in patients with surgery. The results of this study showed no significant difference in DH, LASD, SS, PT, and LL levels between the two groups 3 months after surgery (*p* > 0.05), suggesting that the spinal large-channel endoscopy technology could effectively restore DH, LASD, SS, PT, and LL levels in elderly patients with lumbar spinal stenosis, which was consistent with the results of Li Fuqing et al. [21].

The results of this study suggested that large-channel spinal endoscopy technology can effectively improve the therapeutic efficacy of elderly patients diagnosed with segmental lumbar spinal stenosis. Tu et al.’s study also pointed out that minimally invasive surgery is effective in treating lumbar spinal stenosis with minimal adverse reactions and safety profile [22]. Based on our results, the incidence of adverse reactions in the study group was lower than that in the control group (*p* < 0.05). These findings suggested that large-channel spinal endoscopy technology could effectively reduce the incidence of adverse reactions in elderly patients with segmental lumbar spinal stenosis. In this study, there was one case of lower limb numbness that resolved after postoperative nutritional nerve therapy. Therefore, it is important to formulate a reasonable surgical strategy before surgery to avoid surgical complications. The side with severe symptoms should be selected for the working channel, and the ipsilateral decompression should be performed first, followed by the opposite side. The preservation of the ligamentum flavum before the completion of bone structure treatment can indirectly protect the neural structure. For areas with severe adhesion, floating it can achieve the decompression effect without completely removing it to avoid tearing the dural sac. When rotating the work sleeve and using endoscopic instruments, caution should be exercised to reduce interference with neural tissue. Additionally, our studiers highlights the following advantages of large-channel spinal endoscopic technology in clinical practice: Firstly, the intervertebral approach allows for access to the dorsal side of the nerve root through the lamina intervertebralis, and with the help of the endoscope, the compressive material on the dorsal side of the nerve root can be treated to relieve the compression of the dura and nerve root caused by the cohesive hyperplasia of hypertrophic ligament and articular process. And the clinical application of the intervertebral approach is more convenient, with a relatively flat learning curve and easier to master [23]. Secondly, compared to the traditional lateral approach, the interlaminar approach offers a shorter surgical procedure, reduces soft tissue damage. The angle limitation of the operating channel is smaller, and the intraoperative channel swing angle and amplitude are larger, increasing the decompression range and facilitating contralateral stealth decompression [23]. Thirdly, this surgical approach involves entering the intervertebral space on one side to complete lateral and contralateral stealth decompression, minimizing contralateral surgical damage and preserving the contralateral intervertebral joints and spinous processes. This preservation facilitates the maintenance of lumbar biomechanical stability and reduces the occurrence of iatrogenic lumbar instability [20]. Fourthly, the diameter of the working channel has also been expanded compared to traditional lateral surgery, with an inner diameter of 7.1 mm and an outer diameter of 1 cm, providing more convenience for endoscopic surgery. Additionally, the large-channel posterior endoscopic system is equipped with larger diameter biting forceps and grinding heads, which create conditions for precise, fast, and efficient spinal canal decompression.

In summary, the large-channel spinal endoscopic technology exerted promising results in elderly patients with segmental lumbar spinal stenosis, in terms of reducing the surgical time, intraoperative bleeding, postoperative drainage volume, and hospital stay. The approach also alleviated pain, reduced ODI and JOA scores, and restored lumbar function, with decreased incidence of adverse reactions, thereby promoting patient recovery. However, there were several limitations that should be acknowledged. Considering the short follow-up of our single-center randomized controlled study, future studies with longer follow-ups are warranted to determine the impact of the approach on spinal instability in the long term in treating spinal stenosis.

## Data Availability

The datasets used and/or analyzed during the current study are available from the corresponding author on reasonable request.
